# The Impact of Health Information on the Internet on Health Care and the Physician-Patient Relationship: National U.S. Survey among 1.050 U.S. Physicians

**DOI:** 10.2196/jmir.5.3.e17

**Published:** 2003-08-29

**Authors:** Elizabeth Murray, Bernard Lo, Lance Pollack, Karen Donelan, Joe Catania, Ken Lee, Kinga Zapert, Rachel Turner

**Affiliations:** ^1^Royal Free and University College School of Medicine at University College LondonDepartment of Primary Care and Population SciencesLondonUK; ^2^University of CaliforniaProgram in Medical EthicsSan Francisco CAUSA; ^3^University of CaliforniaHealth Survey Research UnitCenter for AIDS Prevention StudiesSan Francisco CAUSA; ^4^Harvard School of Public HealthDepartment of Health Policy and ManagementBoston MAUSA; ^5^Harris Interactive IncNew York NYUSA

**Keywords:** Physicians, Internet, physician-patient relations

## Abstract

**Background:**

Public use of the Internet for health information is increasing but its effect on health care is unclear. We studied physicians' experience of patients looking for health information on the Internet and their perceptions of the impact of this information on the physician-patient relationship, health care, and workload.

**Methods:**

Cross-sectional survey of a nationally-representative sample of United States physicians (1050 respondents; response rate 53%).

**Results:**

Eighty-five percent of respondents had experienced a patient bringing Internet information to a visit. The quality of information was important: accurate, relevant information benefited, while inaccurate or irrelevant information harmed health care, health outcomes, and the physician-patient relationship. However, the physician's feeling that the patient was challenging his or her authority was the most consistent predictor of a perceived deterioration in the physician-patient relationship (OR = 14.9; 95% CI, 5.5-40.5), in the quality of health care (OR = 3.4; 95% CI, 1.1-10.9), or health outcomes (OR = 5.6; 95% CI, 1.7-18.7). Thirty-eight percent of physicians believed that the patient bringing in information made the visit less time efficient, particularly if the patient wanted something inappropriate (OR = 2.5; 95% CI, 1.5-4.4), or the physician felt challenged (OR = 3.6; 95% CI, 1.8-7.2).

**Conclusions:**

The quality of information on the Internet is paramount: accurate relevant information is beneficial, while inaccurate information is harmful. Physicians appear to acquiesce to clinically-inappropriate requests generated by information from the Internet, either for fear of damaging the physician-patient relationship or because of the negative effect on time efficiency of not doing so. A minority of physicians feels challenged by patients bringing health information to the visit; reasons for this require further research.

## Introduction

An increasing proportion of the public is using the Internet for health information [1]. This is expected to have a "profound effect on medicine" [2], but it is unclear whether this effect will be beneficial or harmful. The advantages of the Internet as a source of health information include convenient access to a massive volume of information, ease of updating information, and the potential for interactive formats that promote understanding and retention of information. Health information on the Internet may make patients better informed, leading to better health outcomes, more appropriate use of health service resources, and a stronger physician-patient relationship [2]. However, health information on the Internet may be misleading or misinterpreted, compromising health behaviors and health outcomes, or resulting in inappropriate requests for clinical interventions [3]. Physicians may accede to inappropriate requests, either because refusal is time consuming, or because they fear refusal would weaken the physician-patient relationship [4,5]. Responding to inappropriate patient requests may be particularly difficult in managed care, where patients may believe that physician refusals may be motivated by the need to control costs [6]. Some physicians may have difficulty adjusting to a more-equal role with patients [7] or may experience conflict with more-assertive patients [8]. There is little information on physicians' experience with patients who have sought health information on the Internet.

We surveyed a nationally-representative sample of physicians about their experience with patients bringing health information from the Internet to office visits. Our aims were to determine physicians' perceptions of the effects of patients bringing health information from the Internet on the physician-patient relationship; time efficiency of the visit; quality of care received by the patient; and patient's health outcomes.

## Methods

### Sample

Two thousand physicians were randomly selected from the national list of physicians provided by the Medical Marketing Service, Inc (MMS). The Medical Marketing Service list is based on the national database of the American Medical Association (AMA) which includes both members and nonmembers of the American Medical Association, and is updated weekly. The American Medical Association database contains over 650000 physicians, and is the most-complete list of physicians available in the United States. Physicians who currently spent over 20 hours a week on direct patient care were included in the survey. The sample was stratified by specialty: primary care, medical specialty, or surgical specialty. Primary care included family practice, general practice, internal medicine, and pediatrics. Ob-Gyn was classified as a surgical specialty.

### Questionnaire

The questionnaire was developed following literature review and focus-group discussions. It was pretested to ensure that the instrument was easy to complete, all areas of interest were covered, and no questions were ambiguous. It consisted of closed-end questions, took approximately 12 minutes to complete, and was in 3 parts. The entire sample received Part 1 of the questionnaire, which elicited general information about views on health information on the Internet and direct-to-consumer advertising (DTCA). Questions included general views on accuracy and effects of such information, and personal use of the Internet at work. Part 2 was sent to a random 50% of the sample, and requested information about the last time a patient brought in information from the Internet. "Last-time" methodology was used to minimize recall bias. Areas explored were the relevance and accuracy of the information, physicians' perceptions of why the patient had brought the information, physicians' responses to the patient, and their views about the impact on health care, health outcomes, and the physician-patient relationship. The other 50% of the sample received a different Part 2, which explored these same areas but with regard to the last time a patient brought in information from direct-to-consumer advertising. The direct-to-consumer advertising data are presented elsewhere [9]. Part 3 was received by the entire sample and obtained demographic and workload information: hours per week on face-to-face consultations, on other tasks related to patient care, and on administrative tasks; numbers of patients seen per week; practice income; proportions of patients on Medicaid, from minority groups, having household incomes of less than $20000 per annum, and without health insurance; geographic setting of practice; age and racial origin of respondent. This was supplemented with information from the Medical Marketing Service database including specialty, year of graduation from medical school, geographic region (East, South, Midwest, West), whether hospital-based or office-based, and whether trained in the United States or overseas.

### Response Rate

Data collection was undertaken between November 2000 and February 2001. The questionnaire was mailed to the selected physicians with a check for US $35 as a token of appreciation for completing the questionnaire. Up to 3 reminders were sent and additional telephone contact made with nonresponders. Of the original 2000 physicians sent the survey, 38 were ineligible because they were deceased, retired, or no longer in practice; and 1050 physicians completed the questionnaire (response rate 53%). Of these, 515 received the Internet version of the questionnaire, and 535 the direct-to-consumer advertising version.

### Analysis

Data were weighted to represent the national population of physicians in the Medical Marketing Service database who spend 20 or more hours per week on direct patient care, using the Medical Marketing Service variables mentioned above. As can be seen in Table 1, there is little difference between weighted and unweighted data, confirming that respondents were representative of US (United States) physicians.

**Table 1:** Demographic, workload, and practice characteristics of respondents

**Demographic and practice characteristics**	**Unweighted No. (%)**			**Weighted No. (%)**		
Age						
<39	222 (22)			198 (20)		
40-49	360 (36)			363 (36)		
50-59	248 (25)			248 (25)		
60+	169 (17)			188 (19)		
Gender						
Female	228 (22)			223 (22)		
Male	808 (78)			812 (78)		
1999 Income from practice						
$100000 or less	177 (19)			179 (19)		
$100001-$150000	298 (31)			297 (31)		
$151001-$200000	194 (20)			195 (20)		
$200001-$250000	128 (13)			126 (13)		
$250001+	162 (17)			160 (17)		
Geographic setting						
Urban	342 (34)			346 (34)		
Suburban	334 (33)			333 (33)		
Small town	275 (27)			273 (27)		
Rural	67 (7)			66 (7)		
Geographic region						
East	288 (27)			298 (28)		
South	316 (30)			310 (30)		
Midwest	231 (22)			230 (22)		
West	215 (21)			213 (20)		
Type of medical specialty						
Primary care	404 (39)			406 (39)		
Medical specialty	350 (33)			355 (34)		
Surgical specialty	296 (28)			289 (28)		
Office-based or Hospital-based						
Office-based	942 (90)			937 (89)		
Hospital-based	108 (10)			113 (11)		
Country of training						
United States	946 (90)			937 (89)		
Foreign	104 (10)			113 (11)		
						
Respondents best estimate of the percentage of their patients who were	**Unweighted Percentiles**			**Weighted Percentiles**		
	**25th**	**50th**	**75th**	**25th**	**50th**	**75th**
Uninsured	3	5	13	3	5	13
On Medicaid	5	10	25	5	10	25
From a minority group	10	20	40	10	20	40
Had an annual household income of $20000 or less	10	15	30	9	15	30
Respondents best estimate of:						
Number of hours spent per week in face-to-face contact with patients	24	32	40	24	32	40
Number of patients seen per week	50	80	105	50	80	104

The analytic approach focused on evaluating univariate and multivariate relationships with 4 clinically-important outcomes — change in physician-patient relationship; time efficiency; quality of care; and patient health outcome — each of which was assessed on a 3-point scale (improved vs no difference vs worsened). All the demographic, workload, and practice variables listed in Table 1 were run against each of these 4 outcome variables. Univariate relationships were calculated using the chi-square statistic or Fisher exact test as appropriate. In addition, univariate relationships were also investigated for an intermediate outcome: whether or not the physician did what the patient requested (yes completely vs yes partially vs no), a variable which in turn is evaluated for its relationship with the 4 main outcome variables.

Although several of the workload and practice characteristics were assessed as continuous variables (eg, percentage of patients who were uninsured, average number of patients seen per week), most were highly skewed, so medians and interquartile ranges are reported for these data. These variables were split at the 75th percentile for analysis of univariate relationships to test for the influence of these factors. This split was chosen over a median split to maximize the opportunity for an effect to be visible.

Separately for each outcome variable, correlates with chi-square statistics achieving *P*< .20 were analyzed using a stepwise multiple-logistic regression procedure to determine the "most-important" correlates, where importance is defined solely by statistical criteria. Each analysis went through several iterations, with each new iteration employing successively more-stringent statistical criteria for inclusion in the model. Each iteration included consideration of a model yielded by a forward-stepwise procedure and a model yielded by a backward-stepwise procedure. Final models include all correlates with a significant ( *P*< .05) or near-significant (.05 < *P*<.10) likelihood ratio test while still achieving adequate fit, operationalized as *P*> .20 on the Hosmer-Lemeshow goodness-of-fit test.

As all data were weighted (except where specified), the appropriate procedures to correct *P* values and standard errors were undertaken. We used the SVYTAB procedure in STATA to obtain the Rao and Scott F-test *P*-values [10], and the SVYLOGIT procedure in STATA to obtain corrected standard errors for parameter estimates.

## Results

### Demographic and Other Characteristics of the Sample

The characteristics of the respondents before and after weighting are presented in Table 1. Weighting made only minimal difference to the characteristics of the sample, confirming that respondents were representative of US physicians. From this point on, all data presented are weighted.

### Personal Use of the Internet

Sixty-one percent (n = 639; 95% CI, 58%-64%) of all respondents used the Internet in their own practice. In this group, the most-frequent uses were to obtain scientific information such as articles or guidelines (88%; 95% CI, 86%-91%) or to e-mail colleagues (63%; 95% CI, 59%-67%). Obtaining clinical information about patients, such as lab results (28%; 95% CI, 25%-32%), and e-mailing patients (16%; 95% CI, 13%-18%) were much less common uses of the Internet by physicians.

### Views About Health Information on the Internet.

Overall, respondents were positive about the recent increase in health information on the Internet, with 75% (95% CI, 72%-77%) of the total sample thinking that it was a good or very-good thing. Only 15% (95% CI, 13%-17%) believed that it was a bad thing, and the remainder were neutral. Similarly, most physicians (77%; 95% CI, 74%-79%) stated that they had encouraged patients to look for information, although only 35% (95% CI, 32%-38%) had referred patients to Web sites.

### Views About Patient Responses to the Internet

Eighty-five percent (95% CI, 82%-87%) of all respondents had experienced an occasion when a patient brought information from the Internet to a visit. For most physicians this is still a relatively-rare event; 59% (95% CI, 56%-62%) of respondents stated that less than one fifth of their patients had done this. 87% (95% CI, 85%-89%) of physicians perceived their patients as being concerned about the quality of information on the Internet, and 84% (95% CI, 82%-86%) of respondents rated their patients as only fair or poor (rather than good, very good, or excellent) at appraising the quality of information on a Web site *.*

### Results From Respondents Whose Patients Brought Health Information on the Internet to a Consultation

#### Last Consultation With a Patient Who Had Brought in Information on the Internet

A random subsample (n = 519) was asked about the last time a patient had brought in health information on the Internet to a consultation and 430 reported that a patient had done so. The remaining data are from these 430 respondents.

#### Quality of Information

Most respondents believed that the last time a patient had brought in health information from the Internet, the information had been very (18%; 95% CI, 15%-22%) or somewhat (64%; 95% CI, 59%-68%) relevant to that patient's problems and very (8%; 95% CI, 5%-11%) or somewhat (66%; 95% CI, 61%-71%) accurate.

#### Reasons for Bringing Information to the Visit and Response to Requests for Interventions

Respondents perceived that the majority of these patients (90%; 95% CI, 87%-93%) had brought them the information because they wanted the physician's opinion on it. Physicians reported that patients sometimes also wanted a change in medication (31%; 95% CI, 27%-36%), a test (26%; 95% CI, 22%-31%), or a referral to a specialist (13%; 95% CI, 10%-17%).

Physicians usually did what the patient wanted, either completely (23%; 95% CI, 19%-28%) or partially (59%; 95% CI, 54%-63%). Univariate associations are shown in Table 2.

**Table 2:** Did you do what the patient wanted?

	**No.**	**Yes, completely %**	**Yes, partially %**	**No %**	***P***
Total	400	23	59	18	
Medical specialty					.004
Surgical specialty	112	29	59	13	
Primary care	152	21	66	14	
Medical specialty	136	22	50	28	
How relevant did you feel the information was to the patient?					.002
Very / somewhat relevant	327	24	61	15	
Not very / not at all relevant	73	19	48	33	
How accurate was the information?					.001<.001
Very / Somewhat	291	27	62	11	
Not very / Not at all	107	14	48	38	
**:** Patient wanted:					.001<.001
Test / Referral / Medication change	184	9	69	22	
Your opinion only	206	37	50	13	
Did you think that the patient's request was not appropriate for their health?					.001<.001
Yes	128	4	59	37	
No	273	32	59	9	
Did you have enough time to discuss the information?					.001<.001
Yes	253	29	53	17	
No	147	13	68	19	
Did you feel the patient was taking responsibility for their health?					.121
Yes	308	25	59	16	
No	89	18	57	24	
Did you feel the patient was challenging your authority?					.001<.001
Yes	69	6	60	34	
No	329	27	58	15	

On multivariate analysis, only 3 factors independently predicted not doing what the patient wanted. Thinking that the patient's request was not appropriate for their health was the most important factor (OR = 4.4; 95% CI, 2.4-8.0), followed by thinking the information that the patient brought in was not accurate (OR = 3.0; 95% CI, 1.6-5.5) and the type of specialty the physician was in. Medical specialists were more likely than primary care physicians and surgical specialists not to do what the patient wanted (for medical specialist compared to primary care physician OR = 2.8; 95% CI, 1.4-5.5, and for medical specialist compared to surgical specialist OR = 2.0; 95% CI, 1.02-4.1).

#### Effect on Physician-Patient Relationship

Most physicians believed that the patient bringing information to the visit had had a beneficial (38%; 95% CI, 33%-43%) or neutral (54%; 95% CI, 49%-59%) effect on the physician-patient relationship. Univariate associations are shown in Table 3.

**Table 3:** Effect on the physician-patient relationship of the patient bringing information from the Internet

	**No.**	**Improved%**	**No difference%**	**Worsened%**	***P***
Total	406	38	54	8	
How relevant did you feel the information was to the patient?					.001<.001
Very / somewhat relevant	331	44	51	5	
Not very / not at all relevant	74	11	66	23	
How accurate was the information?					.001<.001
Very / Somewhat	298	44	52	5	
Not very / Not at all	106	22	59	19	
Did the patient want:					.001<.001
Test / Referral / Medication change	183	36	50	14	
Your opinion only	212	42	55	3	
Did you do what the patient wanted?					.001<.001
Yes, completely	94	53	47	0	
Yes, partially	234	39	55	6	
No	71	15	57	27	
Did you think that the patient request was not appropriate for their health?					.001<.001
Yes	126	27	48	25	
No	280	43	56	1	
Did you have enough time to discuss the information?					.010
Yes	257	40	55	5	
No	148	34	52	14	
Did you feel the patient was taking responsibility for their health?					.001<.001
Yes	313	43	51	6	
No	89	23	62	15	
Did you feel the patient was challenging your authority?					.001<.001
Yes	68	24	40	35	
No	337	41	56	3	

Multivariate analysis yielded 4 factors that were independently associated with a worsening of the physician-patient relationship. The physician feeling that the patient was challenging their authority was the strongest predictor (OR = 14.9; 95% CI, 5.5-40.5) followed by the physician believing that the patient's request was not appropriate for their health (OR = 9.9; 95% CI, 2.7-36.4). Not feeling that the patient was taking responsibility for their health was independently associated with a worsening of the physician-patient relationship (OR = 4.6; 95% CI, 1.7-12.5), as was not doing what the patient wanted (OR = 4.0; 95% CI, 1.7-9.7).

#### Effect on Time Efficiency

Thirty-eight percent (95% CI, 34%-43%) of physicians believed that the effect of the patient bringing information to the consultation harmed their time efficiency while only 16% (95% CI, 13%-20%) believed that it had helped it. Univariate associations are shown in Table 4.

**Table 4:** Effect on time efficiency of the patient bringing information from the Internet to a visit

	**No.**	**Improved%**	**No difference%**	**Worsened%**	***P***
Total	408	16	45	38	
**Workload and practice characteristics:**					
Country of training					.018
United States	376	15	45	40	
Overseas	32	33	46	20	
Proportion of patients on Medicaid					.014
25% or less	307	14	46	40	
> 25%	72	28	44	28	
Number of patients seen per week					.117
100 or fewer	273	18	47	35	
> 100	125	13	41	46	
Did you have enough time to discuss the information?					<.001
Yes	259	19	52	29	
No	148	12	33	55	
**Information characteristics:**					
How relevant did you feel the information was to the patient?					<.001
Very / Somewhat	333	20	47	34	
Not very / Not at all	75	3	40	57	
How accurate was the information?					<.001
Very / Somewhat	299	20	49	31	
Not very / Not at all	108	6	35	58	
**Patient characteristics:**					
Did the patient want:					.087
Test / Referral / Medication change	183	12	44	44	
Your opinion	212	21	46	33	
Did you do what the patient wanted?					<.001
Yes, completely	94	24	48	27	
Yes, partially	233	16	48	36	
No	72	8	33	59	
Did you think that the patient's request was not appropriate for their health?					<.001
Yes	127	12	27	61	
No	281	18	53	28	
Did you feel the patient was taking responsibility for their health?					.016
Yes	315	19	46	35	
No	89	8	43	49	
Did you feel the patient was challenging your authority?					<.001
Yes	69	8	21	71	
No	339	18	50	32	

Multivariate analysis showed that many of these factors were independently associated. Physicians trained in the United States were more likely than physicians trained overseas to feel that time efficiency was worsened (OR = 5.8; 95% CI, 2.0-17.0). Other independently-associated workload factors were not having enough time to discuss the information (OR = 2.6; 95% CI, 1.6-4.3) and seeing over 100 patients per week (OR = 1.8; 95% CI, 1.1-3.0). The physician thinking that the request was not appropriate for the patients health (OR = 2.5; 95% CI, 1.5-4.4), feeling that the patient was challenging their authority (OR = 3.6; 95% CI, 1.8-7.2), or not thinking that the patient was taking responsibility for their health (OR = 2.2; 95% CI, 1.3-3.8) were also independently associated with worsened time efficiency.

#### Effect on Quality of Care

Most physicians believed that the information made no difference to the quality of care the patient received (70%; 95% CI, 66%-74%). More physicians believed that it had been beneficial (25%; 95% CI, 21%-29%) than deleterious (5%; 95% CI, 3%-8%) (Table 5). Logistic regression revealed that the only factor independently associated with a worsening of quality of care was the physician perceiving that the patient was challenging their authority (OR = 3.4; 95% CI, 1.1-10.9).

**Table 5:** Effect of the patient bringing information from the Internet to a visit on quality of care

	**No.**	**Improved%**	**No difference%**	**Worsened%**	***P***
Total	408	25	70	5	
How relevant did you feel the information was to the patient?					<<.001
Very / somewhat relevant	331	29	68	3	
Not very / not at all relevant	75	4	82	14	
How accurate was the information?					<<.001
Very / somewhat accurate	298	29	67	3	
Not very / not at all accurate	108	11	78	11	
Did the patient want:					<<.001
Test / Referral / Medication change	182	22	69	9	
Your opinion	212	28	71	1	
Did you do what the patient wanted?					<<.001
Yes, completely	94	31	68	1	
Yes, partially	232	26	70	4	
No	73	14	72	15	
Did you think that the patient's request was not appropriate for their health?					<<.001
Yes	126	15	71	14	
No	280	29	70	1	
Did you have enough time to discuss the information?					.138
Yes	258	27	69	4	
No	147	20	73	7	
Did you feel the patient was taking responsibility for their health?					.006
Yes	315	28	67	4	
No	89	12	80	8	
Did you feel the patient was challenging your authority?					<<.001
Yes	68	15	68	17	
No	338	26	71	3	

#### Effect on Health Outcomes

Seventy-five percent (95% CI, 71%-79%) of physicians believed that the information had made no difference to the patient's health outcome, 21% (95% CI, 17%-25%) believed that it had improved the health outcome, and only 4% (95% CI, 2%-6%) believed that it had been deleterious (Table 6). On multivariate analysis, only 2 factors were independently associated with the physician's perception of a worsened health outcome: information that was inaccurate (OR = 5.7; 95% CI, 1.6-20.5), or the physician feeling that the patient was challenging their authority (OR = 5.6; 95% CI, 1.7-18.7). Workload and practice characteristics were not associated with effect on health outcomes.

**Table 6:** Effect of the patient bringing information from the Internet to a visit on health outcomes

	**No.**	**Improved%**	**No difference%**	**Worsened%**	***P***
Total	406	21	75	4	
How relevant did you feel the information was to the patient?					<<.001
Very / somewhat relevant	330	25	73	2	
Not very / not at all relevant	75	5	85	10	
How accurate was the information?					<<.001
Very / somewhat accurate	296	26	73	1	
Not very / not at all accurate	107	7	83	10	
Did patient want:					.002
Test / Referral / Medication change	180	20	74	6	
Your opinion	212	23	76	1	
Did you do what the patient wanted?					<<.001
Yes, completely	92	26	72	1	
Yes, partially	232	23	75	2	
No	73	7	80	13	
Did you think that the patient's request was not appropriate for their health?					<<.001
Yes	126	16	74	10	
No	278	23	76	1	
Did you feel the patient was taking responsibility for their health?					.001
Yes	313	24	74	2	
No	89	10	82	8	
Did you feel the patient was challenging your authority?					<<.001
Yes	69	13	74	13	
No	336	22	76	2	

## Discussion

This is the first large nationally-representative sample of physicians to study physician perceptions of the impact of health information on the Internet on quality of health care, health outcomes, health service utilization, and the physician-patient relationship that we could find by searching MEDLINE. We found evidence of both good and bad effects. Our findings have implications for practicing clinicians, policy makers, and researchers.

### Implications

#### The Quality of Online Information is Paramount

Physicians believed that patients bringing in accurate, relevant online information is beneficial and welcomed it. Conversely, physicians believed that inaccurate or irrelevant information harms the quality of care, health outcomes, time efficiency, and the physician-patient relationship. Thus improving the accuracy and relevance of online information available to patients may improve outcomes of interest to health care providers, payers, and consumers. The policy challenge is how to improve the quality of online health information, given the large number of health-related Web sites and the ease with which sites can be updated. Suggestions include "kitemarks" (seals of approval) for quality Web sites, codes of conduct for development and content of Web sites, market forces, directing users to trusted Web sites, filters, rating instruments for users, and public education in evaluating the quality of online information [11- 14]. The effectiveness and practicality of these suggestions remain unproven [15- 18].

#### Responding to Patient Requests for Clinically Inappropriate Interventions

US physicians may feel in a quandary when patients request an inappropriate clinical intervention that they learned about online. Ethically, physicians should refuse inappropriate requests in order to avoid harming the patient and to use health service resources prudently. However, previous studies have suggested that refusing patient requests will reduce patient satisfaction [5,19]. Physicians may be reluctant to jeopardize patient satisfaction because it is used as an index of quality, and can impact on physician income. This dilemma may be particularly acute in managed care, where patients believe that physicians refuse requests on financial grounds rather than clinical grounds [20]. Physicians also perceive that refusing clinically-inappropriate requests is damaging to time efficiency. This perception, or reality, may make physicians unwilling to engage in such discussions, and may, in turn, lead to more inappropriate requests being filled, with subsequent upward pressure on health care costs.

#### Physicians Who Feel Challenged

Seventeen percent of physicians felt that patients were challenging their authority during the visit. This reaction was strongly associated with harms to the physician-patient relationship, quality of care, health outcomes, and time efficiency. Our study cannot determine why physicians feel challenged. Some physicians may be having difficulty adjusting to a more-equal relationship, where the patient has greater access to medical information [7]. Alternatively, some patients may fail to acknowledge the physician's clinical expertise. This is an area for further research.

### Methodological Considerations

Although our response rate is only moderate at 53%, it compares well to other surveys of Internet use by physicians. Because our sample was representative of all US physicians in terms of age, gender, specialty, location of practice, and practice income our results are likely to generalize to all US physicians. In contrast, previous surveys have examined specific branches of medicine [21], used convenience samples [22] or Internet-literate samples [23], had unacceptably-low (21%) response rates [24], or had very-small samples [25]. Response rates in other recent surveys of US physicians are lower than ours [26- 29], and the absence of substantive differences between responders and nonresponders argues against the presence of systematic selection bias.

As with all cross-sectional studies, we cannot determine causality, nor do we have objective data on whether patient requests were truly inappropriate or on quality of care or health outcomes. However, our measures are plausible because physicians address the appropriateness of care and outcomes daily on a professional basis. Patient perceptions of these consultations may have been different, but our results from a population survey of public perceptions of the effects of health information on the Internet are not dissimilar [30].

### Conclusions

Health care organizations, payers, and providers have a strong interest in ensuring both that health information on the Internet is accurate and that physicians have the necessary skills to respond to patients who bring in such information. Vigorous leadership in these areas will be needed if the effect of the Internet on medicine is to be truly beneficial.

## Abbreviations

US: United States
